# The Effect of a Simple and Reproducible Marking Technique on Enhancing Radiation Safety in Surgical Fixation of Proximal Femur Fractures

**DOI:** 10.7759/cureus.86108

**Published:** 2025-06-16

**Authors:** Muhammad Junaid Khan, Ahmed Juanroyee, Hassan Janan, Khawar Waheed, Riaz Mohammed

**Affiliations:** 1 Trauma and Orthopaedics, Gloucestershire Royal Hospital, Gloucester, GBR

**Keywords:** fluoroscopy, fracture fixation, hip fracture, proximal femur fracture, radiation, risk reduction and prevention

## Abstract

Introduction: Proximal femur fractures are among the most common orthopaedic injuries in the UK. Intraoperative fluoroscopy is essential for precise fracture fixation but exposes both patients and surgical teams to ionising radiation, increasing the risk of complications such as cataracts and certain cancers. While various guidelines aim to mitigate these risks, consistent implementation remains a challenge, and there is limited literature describing surgeon-led techniques to directly reduce intraoperative exposure.

Methods: A case-control study was carried out at a single district general hospital in the UK, which performs over 900 hip fracture surgeries annually. The study involved 125 adult patients (aged over 18) diagnosed with AO type A1 and A2 intertrochanteric fractures, who underwent surgical treatment with either a long cephalomedullary femoral nail (Intertan) or a dynamic hip screw (DHS) between July 2020 and January 2025. Data on dose area product (DAP) and screening time were collected from dose reports archived in the Picture Archiving and Communication System (PACS) for procedures performed by two experienced trauma surgeons. The cases were categorised into two groups: one in which the operations were conducted without using the marking technique, and the other where it was applied. The study aims to evaluate the marking method’s impact on radiation exposure by comparing two groups. An observable reduction would underscore its value in enhancing radiation safety and clinical practice.

Results: A significant reduction in both DAP and screening time was observed in the Intertan group using the marking technique. Mean DAP decreased by 45% from 194.47 to 105.65 UGy × m² (p = 0.0001), and mean screening time reduced from 126.30 to 92.12 seconds (p = 0.001), a reduction by 27%. The mean values for DAP and fluoroscopic exposure time were reduced in the control group for both Intertan and DHS procedures; however, the observed reduction was statistically significant only in the Intertan group.

Discussion: The marking technique was effective in reducing radiation exposure during Intertan fixation without compromising surgical efficiency. It is simple, reproducible, and easy to teach, making it particularly useful in training settings and among rotating theatre teams. By streamlining fluoroscopy positioning, the technique promotes better communication with radiographers and supports adherence to the recommended principles of radiation safety. While limited by its single-centre design and small sample size, this study provides early evidence for a practical method of enhancing radiation safety in hip fracture surgery. Further research with larger cohorts is recommended to explore broader applicability and impact on operative efficiency.

## Introduction

Proximal femur fractures are one of the most common injuries in the UK, and approximately 70,000 are reported every year [1]. Fluoroscopy plays a critical role in hip fracture fixation by enhancing surgical precision, and its utilisation has become increasingly prevalent in these procedures. However, this technique poses significant risks due to radiation exposure for both patients and operating staff. Prolonged fluoroscopic exposure has been associated with an elevated risk of ocular complications [2], as well as malignancies of the thyroid, head, and neck regions. [3-6]. The International Agency for Research on Cancer (IARC) has defined ionising radiation as a Group I carcinogen. Patients have been found to have the highest effective dose of radiation in pelvic ring surgeries [7].

These risks to the patient and clinical team are well recognised and widely discussed. The operating surgeon faces a higher risk of radiation exposure compared to the assistant and scrub nurse [8]. Additionally, less experienced surgeons tend to use fluoroscopy more frequently, further increasing their exposure risk. [9,10]. The radiation dose has also been found to be more with long femoral nail compared to dynamic hip screw (DHS) for extracapsular neck of femur fracture [11].

The British Orthopaedic Association (BOA) 2025 guidance on radiation exposure in theatre outlines several protective measures to mitigate risk. These include adherence to the principles of time, distance, and shielding; awareness of scatter radiation; optimal C-arm orientation; avoidance of live screening; proper use of personal protective equipment (PPE); and the 70-degree view for lateral imaging [12]. However, gaps in compliance with the As Low As Reasonably Achievable (ALARA) principle persist, particularly in communication and consistent application of safety protocols [13].

While there’s ample evidence and guidance on the measures to mitigate radiation risk, to our knowledge, no technique has been described in the literature that surgeons can adopt to reduce screening time, exposure time, and radiation dose during proximal femoral fixation surgeries. We have devised a simple and reproducible patient marking technique to be used before surgery when fixing these fractures with either a DHS or a long cephalomedullary femoral nail. Our study compares radiation dose and screening time between two groups of proximal femur fracture patients, with this marking technique applied in one group. The aim is to assess the effectiveness of this technique by comparing the results between the two groups. A demonstrable reduction in these metrics would represent a meaningful advancement in radiation safety and support the clinical value of the technique.

## Materials and methods

A single-centre case-control study was conducted at a high-volume district general hospital in the UK managing over 900 hip fractures annually. All the patients were operated on by two experienced trauma surgeons following the same operative technique.

Inclusion criteria

Data were collected for patients aged >18 years with intertrochanteric fractures (AO/OTA 31-A1 and A2) treated between July 2020 and January 2025. All patients underwent either long cephalomedullary femoral nailing (Smith & Nephew Intertan) or DHS (Smith & Nephew) fixation following standard closed reduction.

Exclusion criteria

Patients under 18 years of age, those with complex 4-part intertrochanteric fractures, cases requiring open reduction, revision procedures, and intracapsular fractures indicated for surgical fixation were excluded.

Intervention and data collection

Both surgeons performed the procedure with (Control) and without (Case) using a simple pre-operative marking technique. All surgeries were performed on a traction table. Both surgeons adhered to identical steps for patient positioning and marking technique.

**Figure 1:**
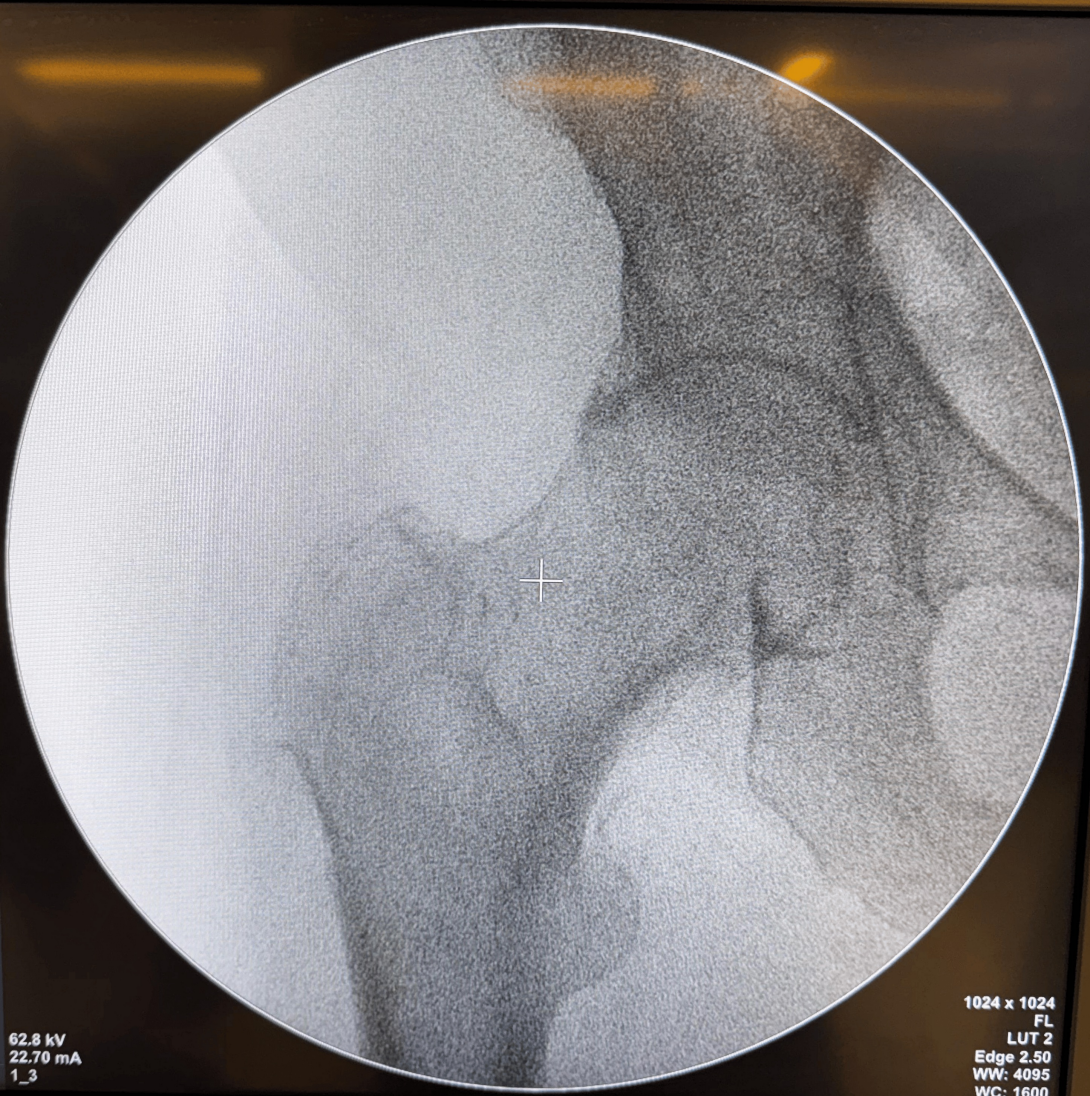
Fluoroscopy image after closed reduction Fluoroscopy image showing fracture reduction after the standard closed reduction technique.

The patient is first positioned on a traction table, and the fracture is reduced using a standard technique of closed reduction (Figure 1).

**Figure 2:**
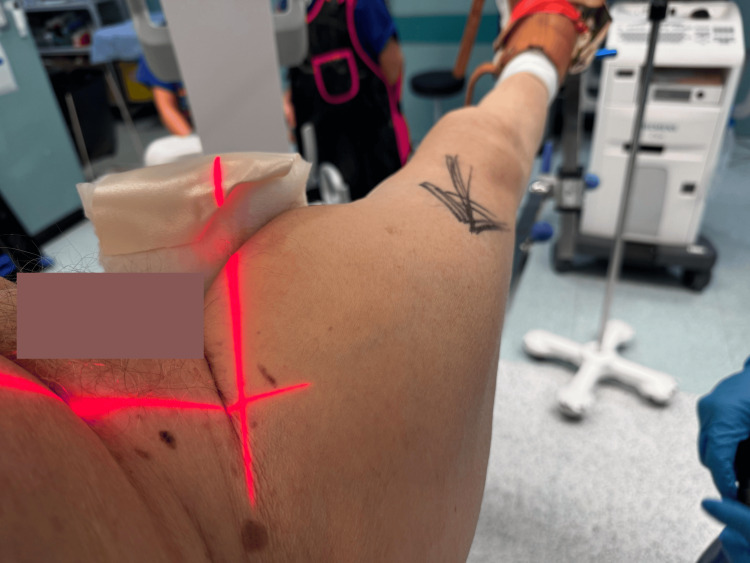
Position of laser mark from II to obtain AP image AP: anteroposterior; II: image intensifier Laser from the II showing the desired position for the AP image.

**Figure 3:**
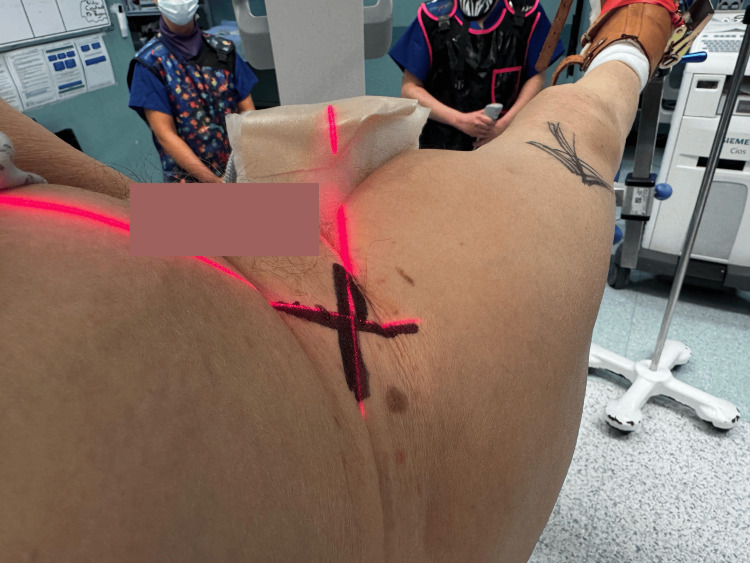
Laser position marked with a permanent marker The laser position is marked with a permanent marker for reference and to guide the radiographer to reposition the image intensifier for subsequent images.

After reduction, the image intensifier (II) is aligned for an anteroposterior (AP) view, with its laser positioned on the hip and marked on the skin using a permanent marker (Figures 2-3).

**Figure 4:**
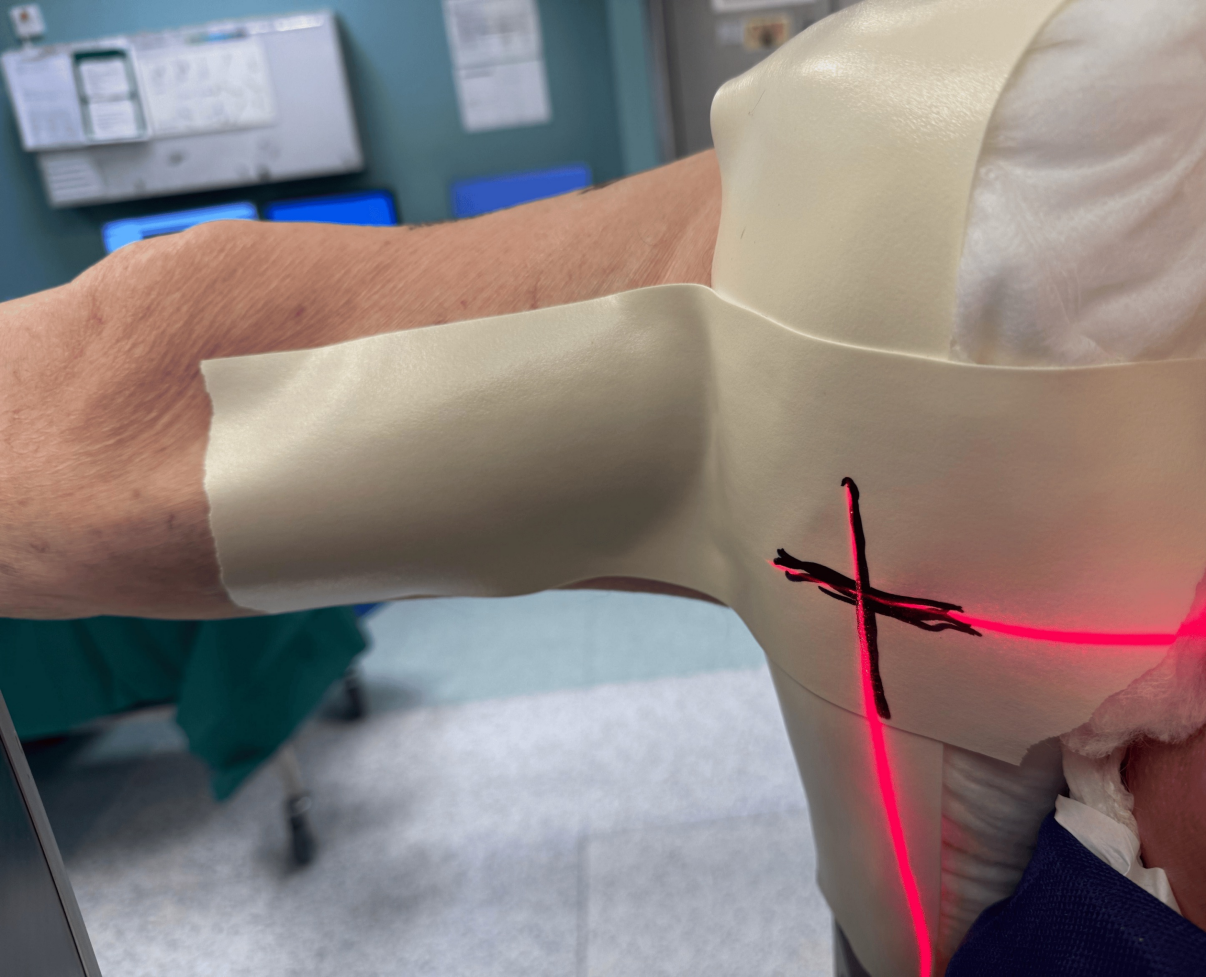
Laser mark position to obtain lateral image Laser position is marked with a permanent marker on Microfoam tape attached to the pelvic post of the traction table to obtain the desired lateral image.

For the lateral view, the II is positioned to obtain the desired lateral image, and the laser position is marked with a permanent marker on Microfoam tape attached to the pelvic post (Figure 4).

**Figure 5:**
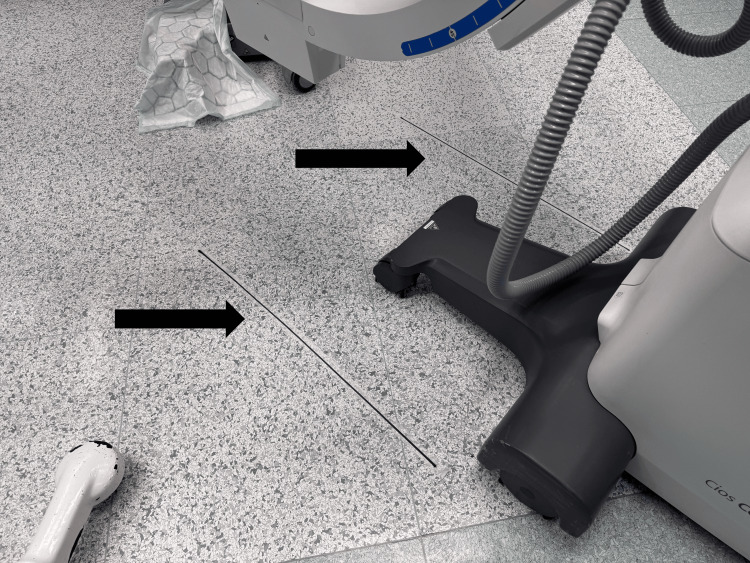
Floor markings to guide image intensifier positioning Dressing tape is attached to the floor on the outer sides of the wheels of the image intensifier (shown with arrows in the figure) to guide its movement for correct positioning.

Dressing tape is stuck to the floor on the outer side of the II’s wheels to guide its positioning during subsequent fluoroscopy (Figure 5).

The exposure to radiation was measured as dose area product (DAP) and exposure or screening time. DAP is calculated by multiplying the dose by the beam area (UGy × m²) and is measured using an ionisation chamber or DAP metre positioned between the X-ray tube/collimator setup and the patient [14]. Exposure or screening time, typically measured in seconds (s) or milliseconds (ms), refers to the duration during which the X-ray tube is active and emitting X-rays to expose both the patient and the image receptor [15]. These measurements were recorded with II (Siemens Healthineers Cios Flow, Germany), and data collected from the dose reports were saved to the Picture Archiving and Communication System (PACS).

The study was carried out as a Quality Improvement Project comparing the effect of the marking technique in cases where it was applied with those without it. It was registered with the Clinical Effectiveness Improvement of our Institution with Log number SG250409.

Statistical analysis

The data was collected and analysed by dividing it into case and control groups for Intertan and DHS. Categorical data was presented as percentages. The data for DAP and screening time were continuous and reported as mean and standard deviation (SD). Comparative analysis of data was performed by applying the Mann-Whitney U test. P-value < 0.05 was considered statistically significant. There were no cases with missing data. GraphPad software (Boston, MA) calculators were used for statistical analysis.

## Results

Data for a total of 125 patients (Female: Male 85: 40) was analysed for the study period. The study group included 68% female and 32% male patients. The Intertan to DHS ratio was 74:51, constituting 59 % and 41% respectively. The mean age for the Intertan group was 83 years (range 62-98 years), whereas that for DHS was 80 years (range 48-101 years). The case group had 41 Intertans and 36 DHS, whereas the control group had 33 Intertans & 15 DHS (Table 1).

**Table 1:** Distribution of patients in both study groups based on age and gender. Table showing the number of patients included in the study, their ages, and gender. They are categorised into those who had surgery with or without the marking technique. DHS: dynamic hip screw

	Without X Mark N (%)	With X Mark N (%)	Total N (%)
Patients (n=125)			
Intertan	41 (53%)	33 (69%)	74 (59%)
DHS	36 (47%)	15 (31%)	51 (41%)
Age in years (mean)			
Intertan	84.94	81.68	83
DHS	79.06	80.13	80
Gender			
Male (n=40)	22 (29%)	18 (37%)	40 (32%)
Female (n=85)	55 (71%)	30 (63%)	85 (68%)

There was a statistically significant reduction in both DAP and screening time for Intertan nailing between the case and control groups, whereas no significant difference was noted for either of them for DHS.

For Intertan, the mean DAP without the marking technique (i.e., case group) was 194.47 UGy × m² (SD - 162.76), whereas it was 105.65 UGy × m² (SD - 105.51) for the control group, showing a 45% reduction. The p-value was calculated to be 0.0001 and hence statistically significant (Table 2).

The mean screening time for Intertan was 126.30 sec (SD - 54.0459) for the case group and 92.12 sec (SD - 47.4703) for the control group, a 27% reduction, which was also statistically significant (p-value = 0.001) (Table 2).

For DHS, on the other hand, the mean DAP was 111.89 UGy × m² (SD - 109.2448) for the case group and 73.69 UGy × m² (SD - 62.8530) for the control group, which was not found to be statistically significant (p-value = 0.23) (Table 2). The mean screening time was 54.71 sec (SD - 25.3021) for the case and 49.87 sec (SD - 30.1825) for the control group, which was also not statistically significant (p-value = 0.45) (Table 2).

**Table 2:** Comparison of Mean dose area product (DAP) and mean radiation time for DHS and Intertan in both study groups This table shows Dose area product (DAP) and screening time for both Intertan and DHS. They are categorised into those who had surgery with and others who had it without the marking technique. DAP: dose area product; SD: standard deviation; DHS: dynamic hip screw

	Without X Mark	With X Mark	p-value
Intertan			
Mean dose area product (uGy m^2^)	194.47	105.65	0.0001
Standard deviation (SD)	162.76	105.51	
Mean radiation time (sec)	126.3	92.12	0.001
Standard deviation (SD)	54.05	47.47	
DHS			
Mean dose area product (uGy m^2^)	111.89	73.69	0.23
Standard deviation (SD)	109.24	62.85	
Mean radiation time (sec)	54.71	49.87	0.45
Standard deviation (SD)	25.31	30.18	

## Discussion

The study found that application of our marking technique was effective in reducing radiation dose and screening time without compromising surgical efficiency. The procedure is easily taught to trainee surgeons as well as to the radiographers operating the fluoroscope. The overall mean values for DAP and exposure time were lower for both Intertan and DHS procedures in the control group. However, this difference was statistically significant only for Intertan. These findings align with existing literature, which similarly reports higher DAP values for long intramedullary femoral nailing compared to DHS. The results are both plausible and clinically relevant, as DHS is a relatively straightforward procedure involving fewer steps requiring image guidance. It is also usually performed for more stable fracture configurations.

Trauma surgeons require enhanced education regarding radiation safety and are not fully versed in optimal fluoroscopy positioning and settings for radiation dose reduction [16]. Our technique streamlines the transition between anterior-posterior and lateral fluoroscopic views, which is frequently required during these procedures. This is particularly relevant for lateral views, as radiation doses are typically higher in this position [17].

Literature supports the experience of radiographers playing a vital role in improving radiation safety [18,19]. However, due to frequently rotating surgical teams, surgeons often face challenges in effectively communicating optimal fluoroscopy positioning requirements to radiographers by using pre-determined markings to guide the placement of the fluoroscope. Our technique minimises the steps needed to optimally position the X-ray tube for acquiring the desired images.

This study demonstrates the effectiveness and practical value of our marking technique and supports this simple, reproducible tool for mitigating radiation risks in proximal femoral fixation surgeries. These findings suggest its potential role in facilitating adherence to ALARA principles of radiation safety. Commonly, these procedures are performed by orthopaedic trainees during the training, and the implementation of a simple, practical technique significantly enhances radiation safety education for junior trainees [20].

The study limitations include its single-centre design, procedures were performed by only two surgeons, and a relatively small patient cohort. We were unable to analyse the technique's effect on overall operative list efficiency. Future research should investigate this approach in a larger patient cohort, particularly when combined with other recommended safety measures. Nevertheless, our study demonstrates merit as a simple, reproducible, and easily teachable technique for promoting radiation safety, with its efficacy supported by statistical findings.

## Conclusions

Long intramedullary femoral nailing leads to greater exposure to radiation and hence the associated risks. The clinical team must be educated about these risks and the information provided to patients. It’s important to adhere to the recommended safety measures and apply such safety techniques in practice to protect both the patient and the operation theatre team. Our simple, reproducible marking technique enables practitioners and radiographers to hone the fluoroscope at the ideal position for a safer and quicker procedure, especially for the cephalomedullary fixation.
